# Bilateral temporal control determines mediolateral margins of stability in symmetric and asymmetric human walking

**DOI:** 10.1038/s41598-019-49033-z

**Published:** 2019-08-29

**Authors:** Tom J. W. Buurke, Claudine J. C. Lamoth, Lucas H. V. van der Woude, At L. Hof, Rob den Otter

**Affiliations:** 1 University of Groningen, University Medical Center Groningen, Center for Human Movement Sciences, Groningen, The Netherlands; 2 University of Groningen, University Medical Center Groningen, Center for Rehabilitation, Groningen, The Netherlands

**Keywords:** Motor control, Biomechanics

## Abstract

Human bipedal gait requires active control of mediolateral dynamic balance to stay upright. The margin of stability is considered a measure of dynamic balance, and larger margins are by many authors assumed to reflect better balance control. The inverted pendulum model of gait indicates that changes in the mediolateral margin of stability are related to changes in bilateral single support times. We propose updated equations for the mediolateral margin of stability in temporally symmetric and asymmetric gait, which now include the single support times of both legs. Based on these equations, we study the relation between bilateral single support times and the mediolateral margin of stability in symmetric, asymmetric, and adaptive human gait. In all conditions, the mediolateral margin of stability during walking followed predictably from bilateral single support times, whereas foot placement co-varied less with the mediolateral margin of stability. Overall, these results demonstrate that the bilateral temporal regulation of gait profoundly affects the mediolateral margin of stability. By exploiting the passive dynamics of bipedal gait, bilateral temporal control may be an efficient mechanism to safeguard dynamic stability during walking, and keep an inherently unstable moving human body upright.

## Introduction

Bipedal gait (as seen in humans) is more energy efficient than quadrupedal gait (as seen in most other mammals)^1^, but to stay upright it also requires active control of mediolateral dynamic balance^2,3^. During standing, the body centre of mass must be within the base of support to remain stable^4^. During walking, more complex control of balance is required, as the vertical projection of the centre of mass is outside of the base of support most of the time. To arrest the fore- and sideward fall in gait, the velocity of the centre of mass should be taken into consideration^5^. To account for the centre of mass velocity next to its position, the concept of the extrapolated centre of mass has been introduced^6^. According to this concept, to remain upright during walking, the extrapolated centre of mass must remain medial to the centre of pressure of the subsequent step^6^. The minimum distance between extrapolated centre of mass and centre of pressure, known as the margin of stability, is considered a measure of dynamic balance and larger margins are sometimes assumed to reflect a better balance control. Maintaining a constant margin of stability is often seen as an important way of balance regulation in both healthy and impaired human gait^7–9^. In the current study, we show that the temporal regulation of gait has profound effects on the mediolateral margin of stability and plays a key role in the production of stable upright bipedal gait.

The concept of the margin of stability is based on the inverted pendulum model of bipedal gait^4,10^. According to this model, centre of mass movements can be conceptualized as motions of a point mass (representing the centre of mass) on top of a single-segment massless pendulum (representing the stance leg), of which lateral oscillations passively depend on gravitational force and the body’s momentum. Bipedal gait can thus be described as a series of controlled falls. Therefore, to understand how humans manage to exploit these falls and stay upright at the same time, we must understand the control of these falling motions.

According to the inverted pendulum model, the mediolateral margin of stability can be modified in two ways. First, through spatial control, i.e. through foot placement or step width regulation^7,9,11^, as a wider step results in a larger mediolateral margin of stability. The second way to change the mediolateral margin of stability is through altering the temporal structure of stepping, i.e. modification of the single support time^8^. When single support time decreases, the inverted pendulum, and therefore the centre of mass, has less time to fall to the side before foot placement. In other words, this reduces the lateral sway of the centre of mass. Therefore, when single support time decreases, the extrapolated centre of mass excursion is smaller, the distance between extrapolated centre of mass and base of support increases, resulting in a larger mediolateral margin of stability. The mediolateral margin of stability can thus be increased by widening the step, but also by decreasing single support time. Multiple studies have assessed foot placement in mediolateral margin of stability regulation^7,9,11,12^, indicating that foot placement after a perturbation (e.g. sideway pushes) can be predicted from the direction and magnitude of centre of mass position and velocity. To the best of our knowledge, the relation between single support time and mediolateral margin of stability has not been assessed systematically, while this relation can be studied simply by exposing humans to different treadmill belt speeds in order to induce changes in single support time.

The inverted pendulum model indicates that the relation between single support time and the mediolateral margin of stability will change once gait becomes asymmetric. As the mediolateral margin of stability at heel-strike partly depends on the preceding contralateral single support time, it follows that the observed mediolateral margin of stability is not only dictated by ipsilateral single support time, but rather by the relation between ipsilateral and contralateral single support times. If in asymmetric gait, the contralateral single support time is reduced, the mean centre of mass and extrapolated centre of mass positions shift towards the ipsilateral side, resulting in an asymmetric mediolateral margin of stability^8,13^. Understanding the relation between bilateral single support times and the mediolateral margin of stability therefore also requires understanding the effect of asymmetric single support times on mediolateral margin of stability. The properties of this bilateral mechanism will not be evident in symmetric gait, when single support times are approximately equal. However, these properties should emerge when people are forced to walk with asymmetric single support times.

Previously^7^, the relation for mediolateral margin of stability *b* has been described as in Eq. 1:

1 $$b=\frac{w}{{e}^{{\omega }_{0}T}+1}$$

with the pendulum eigenfrequency *ω*_0_ = *√g/l*, in which *l* is leg length, *w* is step width and *T* is stance time. From this follows that the mediolateral margin of stability increases with *w* and decreases with *T*.

In the case of asymmetric walking, the respective ipsi- and contralateral mediolateral margin of stability *b*_1_ and *b*_2_ are closely related to *w* and ipsi- and contralateral stance times *T*_1_ and *T*_2_. Based on Hof (2008), for the mediolateral extrapolated centre of mass position ζ we find that:

2 $${{\rm{\zeta }}}_{1}(t)={b}_{1}{e}^{{\omega }_{0}t}\,{\rm{for}}\,0 < t < {T}_{1}$$

3 $${{\rm{\zeta }}}_{2}(t^{\prime} )=w-{b}_{2}{e}^{{\omega }_{0}t\text{'}}\,{\rm{for}}\,0 < t^{\prime}  < {T}_{2}\,{\rm{and}}\,t\mbox{'}=t-{T}_{1}$$

Now it should hold in steady state gait that:

4 $${{\rm{\zeta }}}_{1}({T}_{1})=w-{b}_{2}$$

5 $${{\rm{\zeta }}}_{2}({T}_{2})={b}_{1}$$

Solving Eqs 2 and 3 for conditions 4 and 5 then gives:

6 $${b}_{1}=w\frac{({e}^{{\omega }_{0}{T}_{2}}-1)}{({e}^{{\omega }_{0}({T}_{1}+{T}_{2})}-1)}$$

and

7 $${b}_{2}=w\frac{({e}^{{\omega }_{0}{T}_{1}}-1)}{({e}^{{\omega }_{0}({T}_{1}+{T}_{2})}-1)}$$

It follows that, the leg with the shortest stance time has the largest margin of stability. These equations seem at variance with the expression for symmetric walking in Eq. 1, but if we insert *T*_1_ = *T*_2_ = *T* in Eqs 6 and 7, we find that these new equations are congruent with the expression for symmetric walking (see Eq. 1):

8 $${b}_{1}={b}_{2}=w\frac{({e}^{{\omega }_{0}T}-1)}{({e}^{2{\omega }_{0}T}-1)}=w\frac{({e}^{{\omega }_{0}T}-1)}{({e}^{{\omega }_{0}T}-1)({e}^{{\omega }_{0}T}+1)}=\frac{w}{({e}^{{\omega }_{0}T}+1)}$$

The question remains whether humans actively exploit the relation between bilateral single support time and mediolateral margin of stability to safeguard dynamic stability. To answer this question, purposeful changes in single support time, made in response to a continuous perturbation of steady state gait should result in predictable changes in the mediolateral margin of stability. A suitable paradigm to test this prediction using a continuous perturbation of gait, is split-belt walking. In split-belt walking, people walk faster with one leg than the other. Healthy humans use self-induced changes in single support time^14,15^ and mediolateral margin of stability^13,16^ to adapt to these asymmetric belt speeds within 5–10 minutes^14^. By letting people walk at asymmetric belt speeds, we can induce asymmetric single support times and empirically test the relation between bilateral single support time and mediolateral margin of stability in symmetric, asymmetric, and adaptive gait.

To better understand dynamic balance control during gait, we empirically test whether mediolateral margin of stability and single support time are systematically related (Experiment A – tied belts), and whether the relation between single support time and mediolateral margin of stability depends on bilateral single support times (Experiment B – split belts). In addition, we assess whether changes in bilateral single support time and step width can predict changes in the mediolateral margin of stability during symmetric walking (Experiment A), asymmetric walking (Experiment B) and split-belt adaptation (Experiment C). Step width will be included in all analyses to be able to distinguish the relative contributions of single support time and step width to the mediolateral margin of stability. We predict that single support time and mediolateral margin of stability are closely related, and that this relationship depends on bilateral single support times, as will be observable in asymmetric gait. Furthermore, we hypothesize that the mediolateral margin of stability essentially depends on step width and bilateral single support times, and can be predicted from these parameters in all three experiments.

## Results

### Experiment A – Mediolateral margin of stability and contralateral single support time are strongly related during symmetric walking

**Figure 1:**
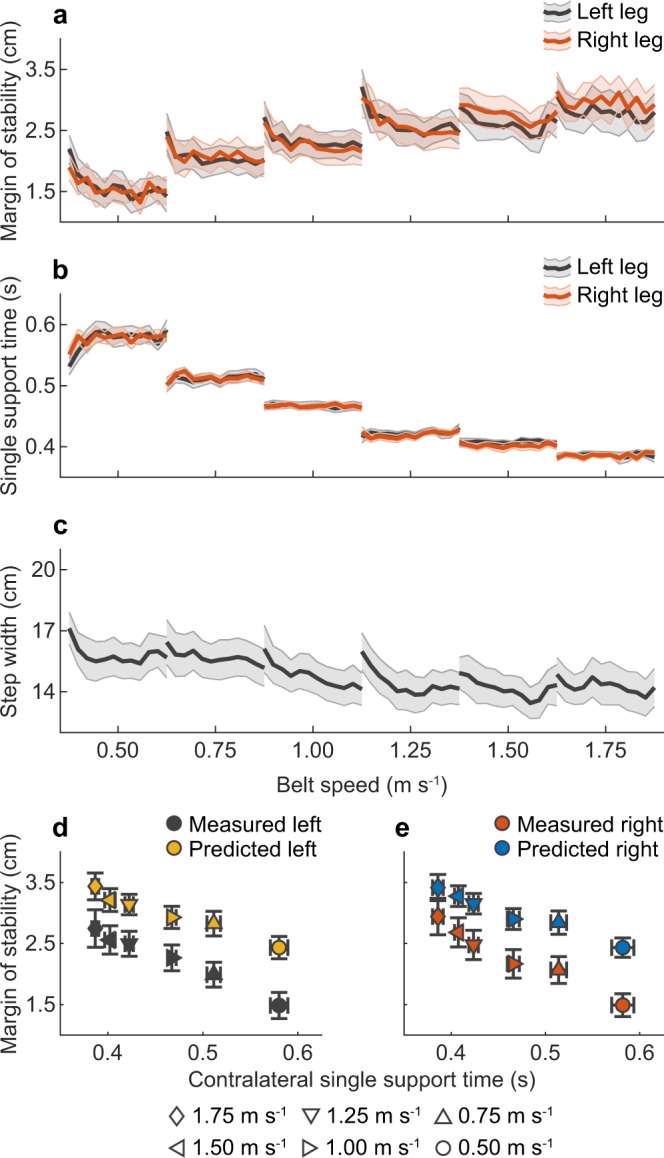
Group-averaged results (N = 15) of Experiment A. This figure illustrates the relationship between margin of stability and single support time in symmetric walking. Margin of stability (**a**), single support times (**b**) and step width (**c**) at symmetric belt speeds ranging from 0.50 m s^−1^ to 1.75 m s^*−*1^. Shaded areas indicate standard error. Measured and predicted left (**d**) and right (**e**) margins of stability vs contralateral single support times. Error bars indicate standard error. Belt speed was scaled to leg length (v*√l)^30^; for interpretation, belt speed for a person with a leg length of 1 m is shown below the x-axis.

In Experiment A we assessed whether single support time and mediolateral margin of stability are related in symmetric walking. Figure 1 shows group-averaged margin of stability (a), single support time (b), step width (c) and measured and predicted group-averaged left (d) and right leg (e) margin of stability vs contralateral single support time. Supplementary Fig. S1 shows step lengths for experiments A, B and C. The average difference ± s.d. between predicted and measured mediolateral margin of stability was 7.4 ± 3.3 mm (left leg) and 7.0 ± 4.4 mm (right leg), indicating a good prediction from the equations, however with an offset (Fig. 1). Furthermore, one-sample Wilcoxon signed-rank tests showed that the median correlation coefficients between left single support time and left margin of stability (median r = −0.94, range = −0.39 – −0.99, Z = −3.408, p < 0.001), right single support time and left margin of stability (median r = −0.92, range = −0.33 – −0.98, Z = −3.408, p < 0.001), left single support time and right margin of stability (median r = −0.93, range = −0.64 – −0.99, Z = −3.408, p < 0.001) and right single support time and right margin of stability (median r = −0.94, range = −0.71 – −0.99, Z = −3.408, p < 0.001) were significantly different from zero. These correlation coefficients imply a very strong relationship between bilateral single support times and mediolateral margin of stability in symmetric walking. In addition, one-sample Wilcoxon signed-rank tests showed that the median correlation between step width and margin of stability was not significantly different from zero for the left (median r = −0.23, range = −0.98 – 0.72, Z = −1.022, p = 0.307) and for the right leg (median r = −0.29, range = −0.97 – 0.73, Z = −1.361, p = 0.173), indicating no relation between step width and margin of stability in symmetric walking.

### Experiment B – The mediolateral margin of stability depends on bilateral single support times during asymmetric walking

**Figure 2:**
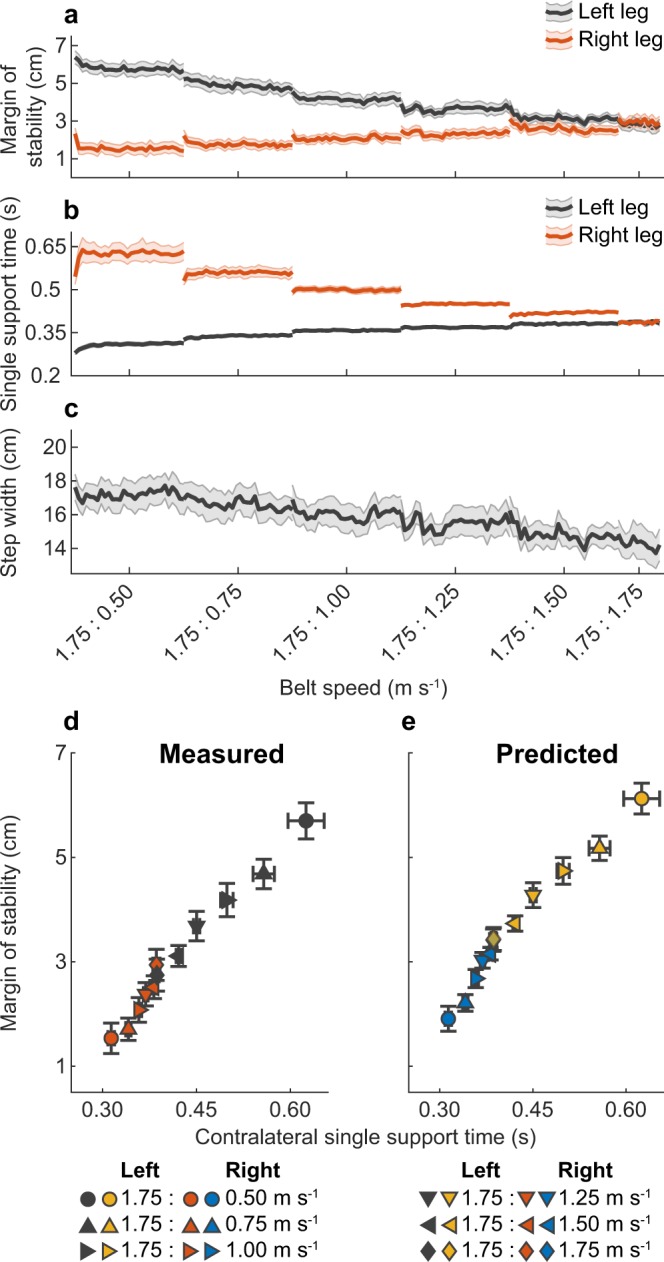
Group-averaged results (N = 15) of Experiment B. This figure illustrates the bilateral relationship between margin of stability and single support time and the good quality of the predictions during asymmetric walking. Margin of stability (**a**), single support times (**b**) and step width (**c**) at asymmetric belt speeds, with the left belt speed set at 1.75 m s^−1^ and right belt speeds ranging from 0.50 m s^−1^ to 1.75 m s^−1^. Shaded areas around the lines indicate standard error. Measured (**d**) and predicted (**e**) left and right leg margin of stability vs contralateral single support time at all asymmetric conditions. Error bars indicate standard error. Belt speed was scaled to leg length (v*√l)^30^; for interpretation, belt speed for a person with a leg length of 1 m is shown below the x-axis.

In Experiment B, we assessed the relation between single support time and mediolateral margin of stability in asymmetric gait, to determine whether the mediolateral margin of stability depends on bilateral single support times. Figure 2 shows group-averaged margin of stability (a), single support time (b), step width (c) and measured (d) and predicted (e) mediolateral margin of stability vs single support time for the left and right leg. The average difference ± s.d. between predicted and measured mediolateral margin of stability was 6.0 ± 3.2 mm (left leg) and 5.5 ± 4.5 mm (right leg), indicating a good prediction from Eqs 6 and 7 in asymmetric walking. Furthermore, one-sample Wilcoxon signed rank tests showed that the median correlation coefficients between left single support time and left margin of stability (median r = −0.95, range = −0.73 – −1.00, Z = −3.408, p < 0.001), right single support time and left margin of stability (median r = 0.95, range = 0.29 – 0.99, Z = 3.408, p < 0.001), left single support time and right margin of stability (median r = 0.88, range = −0.58 – 0.96, Z = 3.237, p = 0.001), and right single support time and right margin of stability (median r = −0.95, range = −0.04 – −0.99, Z = −3.408, p < 0.001) were significantly different from zero. This indicates a strong relation between bilateral single support times and mediolateral margin of stability in asymmetric gait. In addition, one-sample Wilcoxon signed rank tests showed a significant positive relationship between step width and left margin of stability (median r = 0.92, range = 0.31 – 0.98, Z = 3.408, p < 0.001) and negative relationship between step width and right margin of stability (median r = −0.64, range = −0.96 – 0.65, Z = −2.442, p = 0.015) in asymmetric walking, indicating a moderate to strong relationship between step width and mediolateral margin of stability in asymmetric walking.

### Experiment C – The mediolateral margin of stability depends on bilateral single support times and step width during split-belt adaptation

**Figure 3:**
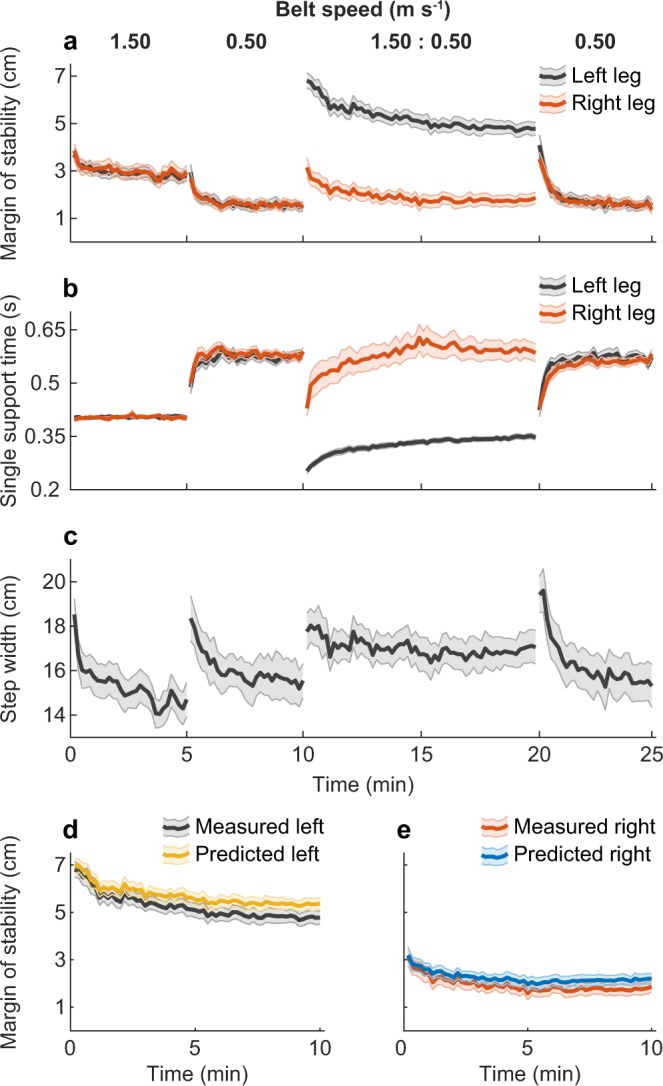
Group-averaged results (N = 15) of Experiment C. Shaded areas around the lines indicate standard error. (**a**) Margin of stability, (**b**) single support times and (**c**) step width during fast (1.50 m s^−1^) and slow (0.50 m s^−1^) baseline gait, split-belt adaptation (1.50: 0.50 m s^−1^) and washout (0.50 m s^−1^). Left (**d**) and right (**e**) leg, measured and predicted margin of stability adaptation curves are shown. Panels D & E indicate that left and right leg margin of stability adaptation curves can be predicted from bilateral single support times and step width. Belt speed was scaled to leg length (v*√l)^30^; for interpretation, belt speed for a person with a leg length of 1 m is shown above panel a.

In Experiment C, we assessed whether humans exploit the relation between single support time and mediolateral margin of stability to safeguard dynamic stability. To this end, mediolateral margin of stability was predicted from bilateral single support times and step width during split-belt adaptation (see Eqs 6 and 7). Figure 3 shows the group-averaged mediolateral margin of stability (a), single support time (b), step width (c) and the observed and predicted bilateral mediolateral margin of stability adaptation curves (d, e). The group-averaged difference ± s.d. between predicted and measured mediolateral margin of stability was 5.0 ± 2.0 mm (left margin of stability) and 4.2 ± 2.8 mm (right margin of stability). This indicates a good prediction of the mediolateral margin of stability and shows that the purposeful changes in bilateral single support times and step width during split-belt adaptation result in predictable changes in the mediolateral margin of stability.

## Discussion

The margin of stability is a well-known measure for dynamic balance control. The inverted pendulum model indicates that changes in the mediolateral margin of stability are related to changes in the temporal regulation of gait, however it is unclear how the margin of stability is regulated. In the present study, we predicted (Eqs 6 and 7) and empirically investigated the relation between single support time, step width and mediolateral margin of stability, in symmetric, asymmetric, and adaptive human gait. The results of three present experiments show that during unperturbed gait on a split-belt treadmill, temporal control has a strong influence on the mediolateral margin of stability. According to theory (Eqs 6 and 7), step width is also a determining factor, but step width was not manipulated in the present study. Furthermore, the mediolateral margin of stability depends on changes in bilateral instead of unilateral single support times. Finally, based on the adjustment of adding bilateral single support times in Eqs 6 and 7 for the mediolateral margin of stability, we were able to accurately predict mediolateral margin of stability from bilateral single support times and step width in symmetric, asymmetric and adaptive split-belt walking.

It follows from the inverted pendulum model that changes in bilateral single support times correspond predictably with changes in mediolateral margin of stability. Our results show that when single support time increases in symmetric gait, the mediolateral margin of stability decreases. Previously, it has been suggested that the mediolateral margin of stability is related to ipsilateral single support time or step frequency^8,17^. However, the new Eqs 6 and 7, and results show that the mediolateral margin of stability depends on both the ipsi- and contralateral, i.e. bilateral, single support times. The addition of bilateral single support times to this model allows a more comprehensive understanding of the control of the mediolateral margin of stability during symmetric and asymmetric human walking. In addition, when people purposefully changed their single support times in response to split-belt adaptation, we accurately predicted changes in mediolateral margin of stability from these bilateral single support times and step width based on Eqs 6 and 7. Therefore, the adaptation of mediolateral margin of stability as reported previously during split-belt walking^13^, can be interpreted as the biomechanical result of changes in the spatiotemporal stepping patterns in gait.

The results show that the mediolateral margin of stability strongly relates to bilateral single support times. Although this behaviour follows directly from the passive properties of the inverted pendulum model, this does not exclude that humans may exploit this relation purposefully to maintain dynamic stability. Humans can intentionally change the temporal structure of gait through, for instance, changes in cadence^18^ or swing speed^15^, thereby regulating balance control. For instance, people tend to make short, quick steps when balance is challenged, e.g. when walking in a moving train or on a rocking boat. Furthermore, active control is always required in response to perturbations, external or self-generated^19^. Active exploitation of these passive properties may be even more prominent in impaired populations. Because even though walking with asymmetric single support times is uncommon and energy-costly in healthy humans^20^, it features prominently in populations with asymmetric leg properties, such as people with a prosthesis or hemiparesis. Humans with problematic stability have possibilities to increase their mediolateral margin of stability: by taking wider steps, or, in the case of asymmetric impairment (e.g. a prosthesis^8^ or hemiparesis^21^), by shortening stance time of the impaired leg. Such an increase margin of stability in itself does not provide better stability, but it gives better opportunities to correct balance, e.g. by action of the unimpaired leg. While people with post-stroke hemiparesis show similar single support times on the non-paretic side as healthy humans at equal gait speeds, they typically show a lower single support time on the paretic side^22^. This may be a direct result of reduction in muscle force production, but can also represent a timing-based strategy to safeguard mediolateral dynamic balance on the paretic side. The latter idea is supported by a study in which it was shown that when people post-stroke lower their gait speed in response to lateral perturbations, they keep their step frequency at the same level as before the perturbation^17^, thereby safeguarding their mediolateral margin of stability. Thus, although the results of the present study show that large variations in the mediolateral margin of stability can be the passive result of changes in the temporal structure of gait, humans might still exploit this relationship to maintain dynamic stability.

Foot placement, as evaluated with step width, played a marginal role in the here observed variations in mediolateral margin of stability during symmetric walking in experiment A. During steady state gait, the possibilities to alter foot placement are limited, as increases in step width may induce higher energy costs associated with step-to-step transitions and lateral limb excursion^23^. In general, foot placement strategies may play a more prominent role when sudden, unexpected perturbations require a balance response under time-critical conditions. Under these circumstances, it may take one or two steps to decelerate the centre of mass through changes in single support time, which is too slow to respond adequately to perturbations such as slips^24^ or pushes^9^. In contrast, deceleration of the centre of mass by means of foot placement is possible within a single step. In the current study, the influence of single support time on the mediolateral margin of stability is highlighted. There is also an effect of step width on the mediolateral margin of stability, but as the experimental results show (Figs 1, 2, 3), step width did not vary a lot in these experimental conditions. To determine the effect of step width on the mediolateral margin of stability, a different experimental set-up is necessary. It is important to note that no discrete perturbations (e.g. pushes or pulls) were applied in the present study, making employment of mediolateral foot placement strategies less likely. In addition, walking on a split belt treadmill, either in tied-belt^25^ or split-belt mode^13^, is associated with large step widths (approximately 17 cm in Experiment C), which limits the possibilities to utilize lateral foot placement for balance modifications and may mask the role of foot placement in this study compared to single-belt treadmill or over-ground walking. Furthermore, in the current study we neglected the role of mediolateral foot roll-off, i.e. centre of pressure displacement within the base of support during single stance^8,13,26^. The mediolateral foot roll-off may explain the small discrepancy between the measured and predicted mediolateral margin of stability in the current study as it defines the mediolateral base of support during single stance. To be able to assess the effect of single support time on the mediolateral margin of stability, the mediolateral margin of stability had to be defined at a discrete point in the gait cycle, in this case the start of the single support. However, it can be expected that the foot roll-off may be actively used to fine-tune deviations from the average margin of stability during stance^8,13,19,26^. In future work this can be studied, to better understand dynamic balance control throughout the gait cycle.

Originally, Hof (2008) suggested that maintaining a constant margin of stability results in stable gait. Although this holds for simulations of gait and would likely result in stable gait in humans as well, our results show that margin of stability constancy is not a requirement for stable human gait, as large variations in mediolateral margin of stability emerge predictably from changes in bilateral single support times during the experiments. Because the mediolateral margin of stability depends on bilateral single support times, inter-limb temporal coordination is crucial for maintaining mediolateral dynamic stability. This temporal regulation may be controlled by relatively simple networks in the spinal cord^27^, so that regulation of dynamic stability through temporal control of gait requires minimal contribution of higher-order supra-spinal control. It can be argued that humans maintain dynamic stability through both temporal and spatial control of gait. The step width is then optimized to an energetic optimum^23^, while changes in single support time are exploited to regulate the mediolateral margin of stability in unperturbed gait.

Human bipedal gait requires active control of mediolateral balance to remain stable^2^ and the (magnitude of the) mediolateral margin of stability is often considered a measure of dynamic stability. This study shows that the mediolateral margin of stability during unperturbed symmetric, asymmetric, and adaptive gait follows predictably from bilateral single support times and step width. The relation between bilateral single support times and mediolateral margin of stability is especially of interest, as it is often neglected and suggests passive regulation of balance, although this relation may still be actively exploited by humans to maintain dynamic stability. These findings should be considered in future studies on dynamic balance control in both healthy and pathological populations. In conclusion, bilateral temporal regulation forms an important mechanism to exploit the passive dynamics of bipedal gait, in order to keep an inherently unstable moving human body upright, with minimal explicit control.

## Methods

Healthy young adults (N = 15, 8 females, mean ± s.d. age: 25.1 ± 2.9 years, weight: 75.7 ± 10.7 kg, height: 1.83 ± 0.08 m) participated in this study. Inclusion criteria were (i) no prior experience with split-belt walking, and (ii) the absence of impairments that affect gait. The ethics committee of the Center for Human Movement Sciences, University Medical Center Groningen approved the experiment protocols of this study (ECB/2018.01.15_1). The methods of this study were carried out in line with the Declaration of Helsinki^28^. All participants gave written informed consent prior to the experiments.

### Instrumentation

An instrumented split-belt treadmill (Motek, Amsterdam, NL) was used in this study. 3D ground reaction forces (N), moments of force (N m) and 2D centre of pressure positions (m) were measured with two embedded force plates and recorded with D-Flow software (Motek, Amsterdam, NL) at 1000 Hz. Data were analysed in an XYZ coordinate system with the x-axis in forward direction, y-axis in vertical direction, and z-axis in left right direction^29^. All data were stored on an encrypted drive for offline analysis. To secure the participants’ safety, they wore a harness that was attached to the ceiling, yet did not provide body weight support or constrain movements. Participants were instructed not to touch the handrails on the sides of the treadmill.

### Experimental protocol

**Figure 4:**
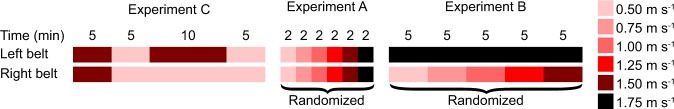
Experimental protocols for Experiments A (symmetric), B (asymmetric) and C (split-belt adaptation). Upper and lower bars show left and right belt speeds. Belt speeds in Experiments A and B were randomized for each participant to reduce the chance of crossover effects. However, participants first completed Experiment A before moving on to Experiment B. Experiment C was measured prior to Experiments A and B to ascertain first-time split-belt adaptation effects. Belt speed was scaled to leg length for each participant.

In Experiments A and B, gait speed was manipulated to induce symmetric and asymmetric single support times, as a change in gait speed leads to spontaneous changes in single support times, whereas manipulating single support times through, e.g. metronome pacing may lead to confounding foot strike timing strategies. In Experiment A, participants walked at six different symmetric belt speeds, ranging from 0.50 m s^−1^ to 1.75 m s^−1^. In Experiment B, participants walked at 5 different asymmetric belt speeds, with the left belt speed fixed at 1.75 m s^−1^ and the right belt speeds ranging from 0.50 m s^−1^ to 1.50 m s^−1^. In Experiment C, participants were exposed to fast (1.50 m s^−1^) and slow (0.50 m s^−1^) tied-belt speeds, a split-belt adaptation phase (1.50: 0.50 m s^−1^) and a slow (0.50 m s^−1^) tied-belt wash-out phase. All conditions are presented in detail in Fig. 4.

In all conditions, belt speed was scaled to leg length by multiplying the nominal speed (v) with the square root of leg length (l)^30^; for interpretation in figures, belt speed is shown for a person with a leg length of 1 m. Experiment C was conducted prior to Experiments A and B, to ascertain first-time exposure to split-belt adaptation in Experiment C, and reduce the time required to adapt to subsequent asymmetric gait conditions in Experiment B. There was no rest between trials, therefore speed conditions were randomized between participants in Experiments A and B to avoid potential effects of fatigue, but Experiment A was always completed in full before continuing with Experiment B. Participants received no instructions regarding the duration of phases or upcoming changes in gait speed, and were instructed to look straight ahead during the experiments.

### Data analysis

All data were analysed off-line in MATLAB (version r2018b; The MathWorks Inc., Natick, MA, USA). Force plate recordings were filtered with a 15 Hz low-pass second-order zero-phase Butterworth filter. Gait events were detected by finding the point at which vertical ground reaction force crossed a threshold of 50 N. Since data was recorded from two force plates, single force plate ground reaction forces were simulated by summation of left and right force plate ground reaction forces. Single force plate centre of pressure data was calculated by scaling the Centre of Pressure (CoP) of each force plate to the magnitude of its respective vertical Ground Reaction Force (GRF_Y_), as shown in Equation 9.

9 $$Co{P}_{combined}=\,\frac{GR{F}_{Y,left}\times \,Co{P}_{left}+\,GR{F}_{Y,right}\times \,Co{P}_{right}}{GR{F}_{Y,left+}GR{F}_{Y,right}}$$

Single support time (s) was defined as the time between contralateral toe-off and contralateral heel-strike, for the left and right leg independently. Step width was defined as the difference between CoP_z_ (m) position at first and second toe-off in a stride, to match the definition of the mediolateral base of support in the mediolateral margin of stability calculation (see below). The mediolateral centre of mass (CoM) position (m) was calculated by combining the 1) two times high-pass filtered horizontal acceleration of the centre of mass (m s^−2^) and 2) the low-pass filtered CoP_z_ position (m)^13,31,32^, described in more detail in Buurke *et al*. (2018)^13^. The mediolateral Extrapolated Centre of Mass (XCoM) position was then calculated as in Equation 10:

10 $$XCoM=CoM+\frac{vCoM}{\sqrt{g/l}}$$

in which *l* is leg length (m), defined as greater trochanter height multiplied by 1.2^6^. The mediolateral margin of stability (m) was defined as the distance between the mediolateral extrapolated centre of mass position (m) and CoP_z_ position (m) at contralateral toe-off, and calculated for the left and right leg independently^6,13^.

### Statistical analysis

Statistical analysis was performed with the MATLAB Statistics and Machine Learning Toolbox (version r2018b; The MathWorks Inc., Natick, MA, USA). Statistical significance was set at an alpha of 5% for all analyses. For data from Experiments A and B, single support time, step width and mediolateral margin of stability were averaged over the last minute of each condition. The final phase of Experiment A (1.75 m s^−1^ tied-belt) was also used in the analysis of Experiment B to acquire a full range of asymmetric to symmetric belt speeds. For data from Experiment C, single support time, step width and mediolateral margin of stability were averaged into bins of ten seconds.

In Experiments A and B, we examined the relation between single support time and mediolateral margin of stability, and between step width and mediolateral margin of stability. To this end, a Pearson correlation coefficient between single support time and mediolateral margin of stability, and step width and mediolateral margin of stability was calculated for every participant, and for the left and right leg independently, as a score for the relation between these parameters. Then, one-sample Wilcoxon signed-rank tests were used to non-parametrically test whether the group median correlation coefficient was different from zero.

In Experiments A, B and C, we studied whether changes in single support time resulted in predictable changes in mediolateral margin of stability based on the proposed Equations (6 and 7). To this end, individual participant’s bilateral single support times and step width from these experiments and the corresponding participant’s leg length were entered into Equations 6 and 7. This resulted in a predicted left and right mediolateral margin of stability for each participant and experiment. To quantify prediction quality, group-averaged differences between predicted and measured mediolateral margin of stability were calculated for the left and right leg, in all experiments.

## Supplementary information

Supplementary information

Dataset 1

## Data Availability

All data generated or analysed during this study are included in this published article and its Supplementary Information files.

## References

[1] Alexander RM (2004). Bipedal animals, and their differences from humans. J. Anat..

[2] Bauby CE, Kuo AD (2000). Active control of lateral balance in human walking. J. Biomech..

[3] Kuo AD, Donelan JM (2010). Dynamic principles of gait and their clinical implications. Phys. Ther..

[4] Winter DA (1995). Human balance and posture control during standing and walking. Gait Posture.

[5] Pai YC, Patton J (1997). Center of mass velocity-position predictions for balance control. J. Biomech..

[6] Hof AL, Gazendam MGJ, Sinke WE (2005). The condition for dynamic stability. J. Biomech..

[7] Hof AL (2008). The ‘extrapolated center of mass’ concept suggests a simple control of balance in walking. Hum. Mov. Sci..

[8] Hof AL, van Bockel RM, Schoppen T, Postema K (2007). Control of lateral balance in walking. Experimental findings in normal subjects and above-knee amputees. Gait Posture.

[9] Hof AL, Vermerris SM, Gjaltema WA (2010). Balance responses to lateral perturbations in human treadmill walking. J. Exp. Biol..

[10] Geursen JB, Altena D, Massen CH, Verduin M (1976). A model of the standing man for the description of his dynamic behaviour. Agressologie.

[11] Vlutters M, van Asseldonk EHF, van der Kooij H (2016). Center of mass velocity-based predictions in balance recovery following pelvis perturbations during human walking. J. Exp. Biol..

[12] Wang Y, Srinivasan M (2014). Stepping in the direction of the fall: the next foot placement can be predicted from current upper body state in steady-state walking. Biol. Lett..

[13] Buurke TJW, Lamoth CJC, Vervoort D, van der Woude LHV, den Otter R (2018). Adaptive control of dynamic balance in human gait on a split-belt treadmill. J. Exp. Biol..

[14] Reisman DS, Block HJ, Bastian AJ (2005). Interlimb coordination during locomotion: what can be adapted and stored?. J. Neurophysiol..

[15] Vervoort D (2019). Effects of Aging and Task Prioritization on Split-Belt Gait Adaptation. Front. Aging Neurosci..

[16] Park, S. & Finley, J. M. Characterizing dynamic balance during adaptive locomotor learning. *EMBC* (2017). 10.1109/EMBC.2017.8036760 29059808

[17] Hak L (2013). Stepping strategies used by post-stroke individuals to maintain margins of stability during walking. Clin. Biomech..

[18] Hak L, Houdijk H, Beek PJ, van Dieen JH (2013). Steps to take to enhance gait Stability: The effect of stride frequency, stride length, and walking speed on local dynamic stability and margins of stability. PLoS ONE.

[19] Hof AL, Duysens J (2013). Responses of human hip abductor muscles to lateral balance perturbations during walking. Exp. Brain Res..

[20] Ellis RG, Howard KC, Kram R (2013). The metabolic and mechanical costs of step time asymmetry in walking. Proc. R. Soc. B..

[21] Vistamehr A, Kautz SA, Bowden MG, Neptune RR (2016). Correlations between measures of dynamic balance in individuals with post-stroke hemiparesis. J. Biomech..

[22] Lehmann JF, Condon SM, Price R, Delateur BJ (1987). Gait abnormalities in hemiplegia: their correction by ankle-foot orthoses. Arch. Phys. Med. Rehabil..

[23] Donelan JM, Kram R, Kuo AD (2001). Mechanical and metabolic determinants of the preferred step width in human walking. Proc. R. Soc. B..

[24] Bhatt T, Wening JD, Pai Y (2006). - Adaptive control of gait stability in reducing slip-related backward loss of balance. Exp. Brain. Res..

[25] Zeni JA, Higginson JS (2010). Gait parameters and stride-to-stride variability during familiarization to walking on a split-belt treadmill. Clin. Biomech..

[26] Reimann H (2017). Complementary mechanisms for upright balance during walking. PLoS ONE.

[27] Duysens J, Van de Crommert HWAA (1998). Neural control of locomotion; Part 1: The central pattern generator from cats to humans. Gait Posture.

[28] World Medical Association (2013). World Medical Association declaration of Helsinki: ethical principles for medical research involving human subjects. JAMA.

[29] Wu G, Cavanagh PR (1995). ISB recommendations for standardization in the reporting of kinematic data. J. Biomech..

[30] Hof AL (1996). Scaling gait data to body size. Gait Posture.

[31] Schepers HM, van Asseldonk EHF, Buurke JH, Veltink PH (2009). Ambulatory estimation of center of mass displacement during walking. IEEE Trans. Biomed. Eng..

[32] Hof AL (2005). Comparison of three methods to estimate the center of mass during balance assessment. J. Biomech..

