# Supplementary material for: Bilateral temporal control determines mediolateral margins of stability in symmetric and asymmetric human walking
Source: Sci Rep. 2019 Aug 29;9:12494. doi: 10.1038/s41598-019-49033-z (PMC6715793; doi:10.1038/s41598-019-49033-z)
Supplement: Supplementary file 1 — Supplementary information [file 41598_2019_49033_MOESM1_ESM.pdf]

Supplementary information

Bilateral temporal control determines mediolateral margins of stability in symmetric and asymmetric human walking

Tom J.W. Buurke[1*], Claudine J.C. Lamoth[1], Lucas H.V. van der Woude[1,2], At L. Hof[1], Rob den Otter[1]

[1]University of Groningen, University Medical Center Groningen, Center for Human Movement Sciences, Groningen, The Netherlands

[2]University of Groningen, University Medical Center Groningen, Center for Rehabilitation, Groningen, The Netherlands

*Corresponding author email: T.J.W.Buurke@umcg.nl

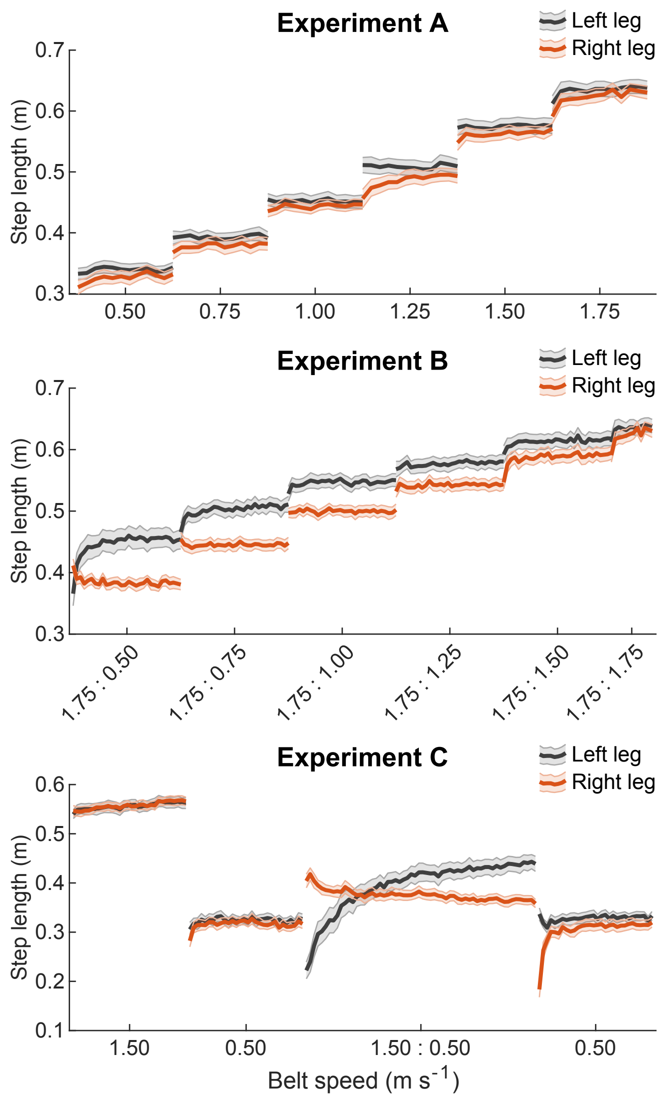

Supplementary Figure S1 - Group-averaged step lengths (N=15) of all experiments. Shaded areas around the lines indicate standard error.
